# Oncocytoma Arising From an Accessory Adrenal Gland: A Case Report

**DOI:** 10.1002/iju5.70245

**Published:** 2026-08-07

**Authors:** Yuki Seki, Kento Morozumi, Naomi Sato, Masaaki Oikawa, Hiroki Kusumoto, Nao Iwamoto, Naoki Kawamorita, Takayuki Yamada, Yasuhiro Nakamura, Yasuhiro Kaiho

**Affiliations:** ^1^ Department of Urology Tohoku Medical and Pharmaceutical University Hospital Sendai Japan; ^2^ Department of Pathology Tohoku Medical and Pharmaceutical University Hospital Sendai Japan; ^3^ Department of Radiology Tohoku Medical and Pharmaceutical University Hospital Sendai Japan

**Keywords:** accessory adrenal gland, adrenal rests, Lin–Weiss–Bisceglia criteria, oncocytoma, steroidogenic factor 1

## Abstract

**Introduction:**

Accessory adrenal oncocytomas are rare. Consequently, the clinical characteristics and management of this entity remain poorly defined.

**Case Presentation:**

A 65‐year‐old man presented with a retroperitoneal mass. Imaging revealed a 103 × 96 mm tumor adjacent to the left kidney. Hormonal evaluation indicated the tumor was nonfunctioning; however, dehydroepiandrosterone sulfate and neuron‐specific enolase levels were elevated, and malignancy could not be excluded. The tumor was resected laparoscopically. Histopathological examination revealed oncocytic features, and immunohistochemistry demonstrated positivity for steroidogenic factor 1. The tumor was anatomically separate from the normal adrenal gland, supporting the diagnosis of an accessory adrenal oncocytoma. Based on the Lin–Weiss–Bisceglia criteria, the tumor was classified as borderline malignant potential. No recurrence has been observed during 2 years of follow‐up.

**Conclusion:**

Accessory adrenal oncocytomas are rare entities, and their accurate diagnosis requires a comprehensive, integrated approach incorporating clinical findings, imaging studies, and detailed pathological evaluation.

## Keynote Message

Accessory adrenal oncocytoma is an extremely rare tumor that can mimic adrenal or retroperitoneal malignancy. Because no specific imaging or endocrine features have been established, preoperative diagnosis is often difficult. In this case, steroidogenic factor 1 immunohistochemistry and the Lin–Weiss–Bisceglia criteria were essential for accurate diagnosis and risk assessment.

## 1 Introduction

Oncocytomas are histologically characterized by abundant eosinophilic granular cytoplasm and most commonly arise in the salivary glands, kidneys, thyroid glands, parathyroid glands, and pituitary gland, whereas occurrence in the adrenal gland is rare [1]. When additional adrenal tissue is present alongside a normally positioned adrenal gland, it is referred to as an accessory adrenal gland [2]. Oncocytomas arising in the adrenal gland are rare [3, 4], and those originating from accessory adrenal glands are exceedingly uncommon.

Herein, we report a rare case of accessory adrenal oncocytoma in which immunohistochemistry (IHC), particularly steroidogenic factor 1 (SF‐1), and the Lin–Weiss–Bisceglia (LWB) criteria were useful for diagnosis [5, 6].

## 2 Case Presentation

A 65‐year‐old man was referred to our department for evaluation of a retroperitoneal mass detected on abdominal ultrasonography during assessment of worsening glycemic control.

**FIGURE 1:**
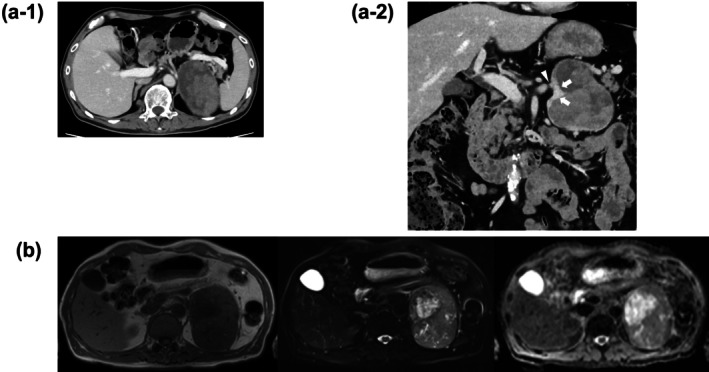
Contrast‐enhanced computed tomography (CT) and magnetic resonance imaging (MRI) findings of the tumor. (a1) Contrast‐enhanced CT showing a mass located superior and anterior to the left kidney, with medial displacement of the left adrenal gland by the tumor. (a2) Coronal contrast‐enhanced CT of the retroperitoneal tumor. The tumor is located adjacent to the left adrenal gland. The arrows indicate the compressed left adrenal gland, and the arrowhead indicates the left adrenal vein. (b) Contrast‐enhanced MRI. From left to right: T1‐weighted imaging (T1WI), T2‐weighted imaging (T2WI), and apparent diffusion coefficient (ADC) map. The tumor demonstrates signal intensity comparable to that of the renal parenchyma on T1WI and T2WI, and slightly lower signal intensity than the kidney on the ADC map.

Contrast‐enhanced computed tomography (CT) revealed a 103 × 96 mm tumor located superior and anterior to the left kidney, with medial displacement of the left adrenal gland (Figure 1a). Magnetic resonance imaging (MRI) demonstrated a heterogeneous mass with gradual enhancement and restricted diffusion (Figure 1b). Based on its anatomical location and vascular anatomy, an adrenal tumor or primary retroperitoneal mesenchymal tumor was suspected. Hormonal evaluation showed no evidence of functional adrenal hormone secretion; however, elevated dehydroepiandrosterone sulfate (DHEA‐S: 424 μg/dL) and neuron‐specific enolase (NSE: 26.7 ng/mL) raised concern for malignancy (Table 1). Therefore, laparoscopic tumor resection was performed.

**TABLE 1:** Tumor markers and endocrine findings.

Parameter	Value	Unit
Tumor markers		
AFP	1.8	ng/mL
PIVKA‐II	16	mAU/mL
PSA	1.04	ng/mL
SCC	1.1	ng/mL
NSE	26.7	ng/mL
CYFRA	2.8	ng/mL
sIL‐2R	214	U/mL
ProGRP	38.4	pg/mL
Endocrine findings		
DHEA‐S	424	μg/dL
Plasma renin activity (PRA)	1.1	ng/mL/h
Aldosterone	46.4	pg/mL
ACTH	26.8	pg/mL
Cortisol	10.2	μg/dL
Adrenaline	19	pg/mL
Noradrenaline	107	pg/mL
Dopamine	38	pg/mL
Metanephrine	0.27	mg/L
Normetanephrine	0.29	mg/L

**FIGURE 2:**
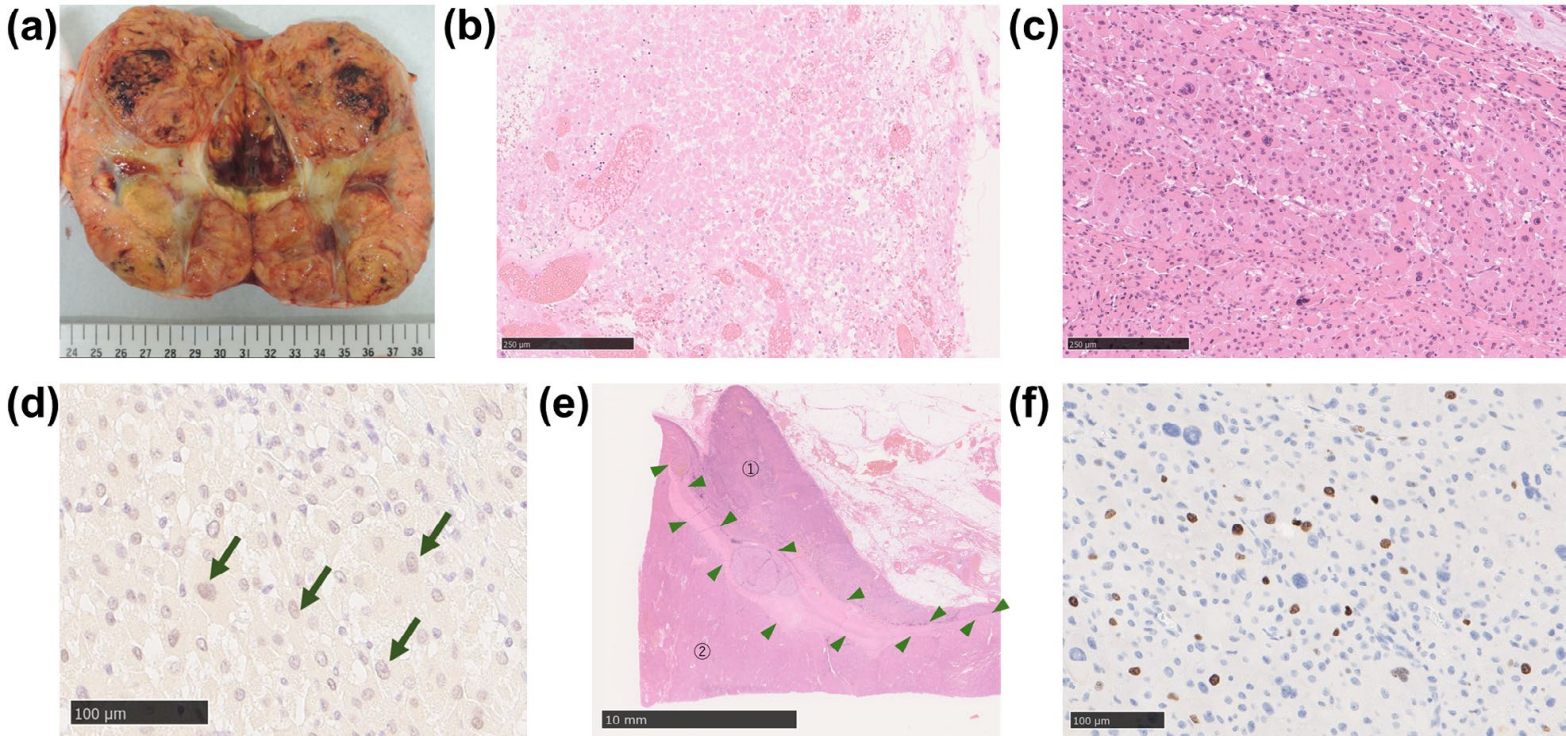
Pathological findings of the resected tumor. Pathological findings of the resected tumor. (a) Gross appearance showing a yellow nodular tumor with focal necrosis. (b) Histological evidence of focal tumor necrosis. (c) Hematoxylin and eosin (H&E) staining demonstrating tumor cells with abundant eosinophilic cytoplasm. (d) Immunohistochemical staining (IHC) for steroidogenic factor 1 (SF‐1) showing nuclear positivity. (e) Low‐magnification H&E staining showing normal adrenal tissue (1) adjacent to the tumor (2). A well‐defined fibrous septum (arrowheads) demarcates the tumor from the normal adrenal gland, supporting their anatomical separation. (f) IHC for Ki‐67. Ki‐67 immunostaining demonstrates nuclear positivity in a subset of tumor cells, with a labeling index of approximately 8.5%, indicating moderate proliferative activity.

The resected specimen weighed 483 g and showed a well‐circumscribed yellow‐tan tumor with focal necrosis on the cut surface (Figure 2a,b). Histological examination revealed the tumor was composed predominantly of oncocytic cells with abundant eosinophilic granular cytoplasm and round to mildly pleomorphic nuclei, showing a diffuse growth pattern without a spindle cell component (Figure 2c). Marked nuclear atypia was observed, consistent with a characteristic feature of adrenocortical oncocytic neoplasms. Immunohistochemically, the tumor showed diffuse positivity for SF‐1, confirming adrenocortical differentiation (Figure 2d). Residual adrenocortical tissue was identified within the lesion, and the tumor was anatomically distinct from the adjacent adrenal gland (Figure 2e).

Although the tumor would have been classified as malignant according to the Weiss criteria, application of the Lin–Weiss–Bisceglia criteria resulted in a diagnosis of borderline malignant potential because only two minor criteria, tumor size > 10 cm and focal necrosis, were present. The Ki‐67 labeling index was 8.5% (Figure 2f). Postoperatively, DHEA‐S and NSE levels decreased (DHEA‐S: 216 μg/dL; NSE: 4.2 ng/mL), and no recurrence has been observed during 2 years of follow‐up.

## 3 Discussion

Preoperative diagnosis of oncocytoma arising from accessory adrenal tissue remains challenging because no characteristic imaging features have been established. Therefore, imaging alone is insufficient for a definitive diagnosis, and integration of clinical, endocrine, and pathological findings is required.

Establishing a definitive preoperative diagnosis based on serum biomarkers is also difficult, as adrenal oncocytomas are typically nonfunctioning, with hormonally active tumors reported in approximately 17% of cases [7]. In the present case, serum catecholamine levels were within normal limits, making pheochromocytoma unlikely, whereas elevated DHEA‐S and NSE levels raised concern for malignancy. Both markers decreased following tumor resection, suggesting an association with the tumor. While elevated DHEA‐S levels may support an adrenocortical origin, the clinical significance of NSE elevation remains unclear and has rarely been discussed in adrenal oncocytic neoplasms [8]. One possible explanation is that oncocytic tumors contain abundant mitochondria, which may reflect increased cellular metabolic activity and contribute to nonspecific NSE elevation. However, this hypothesis remains speculative and requires further investigation.

IHC findings, particularly SF‐1, played a crucial role in establishing the diagnosis. SF‐1 is a highly sensitive and specific marker of adrenocortical differentiation and is useful for distinguishing adrenocortical tumors from morphologically similar oncocytic neoplasms [6]. In the present case, preoperative CT demonstrated an intact left adrenal gland separate from the tumor. Pathological examination showed diffuse SF‐1 positivity and residual adrenocortical tissue within the lesion, while a fibrous septum clearly separated the tumor from the normal adrenal gland. Together, these findings supported the diagnosis of an oncocytoma arising from an accessory adrenal gland.

According to the LWB criteria, the tumor in this case was classified as an accessory adrenal oncocytoma with borderline malignant potential. Compared with the Weiss criteria, which may overestimate malignancy in oncocytic tumors, the LWB system provides a more appropriate assessment of biological behavior [4]. From a clinical perspective, tumors classified as having borderline malignant potential often require careful management and postoperative surveillance comparable to that for malignant tumors. However, recent studies have suggested that even the LWB criteria may overestimate malignant potential in certain adrenal oncocytomas. In the present case, only two minor LWB criteria—large tumor size and focal necrosis—were fulfilled, and the Ki‐67 labeling index was approximately 8.5%, without evidence of highly aggressive biological behavior. Therefore, postoperative surveillance with contrast‐enhanced CT every 6 months during the first postoperative year and annually thereafter was performed, and no evidence of recurrence has been observed during 2 years of follow‐up.

This case represents a rare oncocytoma arising from an accessory adrenal gland. Accessory adrenal glands result from aberrant migration of adrenal cortical tissue during embryogenesis, explaining their typical retroperitoneal location [9]. Adrenal oncocytomas are rare, with approximately 287 cases reported worldwide [4]. Furthermore, accessory adrenal oncocytomas are exceedingly rare; to the best of our knowledge, only 13 cases, including the present case, have been reported in the literature (Table 2) [9, 10, 11, 12, 13, 14, 15, 16, 17, 18, 19, 20]. Further accumulation of cases is necessary to clarify its clinicopathological features and establish appropriate diagnostic and management strategies (Table S1).

**TABLE 2:** Reported PubMed‐indexed cases of the oncocytoma arising from an accessory adrenal gland.

Case	Year	Age (years)	Sex	Country	Tumor location	Malignant potential	Surgical approach
1	1992	44	Male	Canada	Retroperitoneum	Benign	Not described
2	2002	40	Female	Italy	Retroperitoneum	Benign	Not described
3	2004	27	Male	USA	Intraspinal	Benign	Spinal surgery
4	2009	44	Female	Germany	Intraspinal	Benign	Spinal surgery
5	2012	55	Female	USA	Retroperitoneum	Benign	Not described
6	2014	29	Female	Japan	Uterus	Benign	Not described
7	2014	65	Female	Germany	Left kidney	Benign	Laparoscopic
8	2017	2	Male	Spain	Spinal canal	Benign	Spinal surgery
9	2017	Not described	Not described	Korea	Liver	Benign	Not described
10	2019	56	Male	Turkey	Retroperitoneum	Benign	Not described
11	2020	26	Female	USA	Retroperitoneum	Malignant	Open
12	2021	44	Male	China	Liver	Benign	Partial hepatectomy
Present case	2026	65	Male	Japan	Retroperitoneum	Borderline malignant	Laparoscopic

## 4 Conclusion

Accessory adrenal oncocytomas are extremely rare, and SF‐1 immunohistochemistry together with the LWB criteria is valuable for diagnosis.

## Ethics Statement

This study was approved by the ethics review board of Tohoku Medical and Pharmaceutical University (approval number: 2017‐2‐044‐2024).

## Consent

Written informed consent was obtained from patient for publication of this case report and accompanying images.

## Conflicts of Interest

The authors declare no conflicts of interest.

## Supporting information


**Table S1:** Differential diagnosis of peri‐adrenal and peri‐renal masses.

## Data Availability

The data that support the findings of this study are available on request from the corresponding author. The data are not publicly available due to privacy or ethical restrictions.
